# Texture and Friction Classification: Optical TacTip vs. Vibrational Piezoeletric and Accelerometer Tactile Sensors

**DOI:** 10.3390/s25164971

**Published:** 2025-08-11

**Authors:** Dexter R. Shepherd, Phil Husbands, Andrew Philippides, Chris Johnson

**Affiliations:** AI Group, Department of Informatics, University of Sussex, Brighton BN1 9RH, UK; andrewop@sussex.ac.uk (A.P.); c.a.johnson@sussex.ac.uk (C.J.)

**Keywords:** optical tactile sensing, electrical tactile sensing, texture classification, sensor resolution

## Abstract

Tactile sensing is increasingly vital in robotics, especially for tasks like object manipulation and texture classification. Among tactile technologies, optical and electrical sensors are widely used, yet no rigorous direct comparison of their performance has been conducted. This paper addresses that gap by presenting a comparative study between a high-resolution optical tactile sensor (a modified TacTip) and a low-resolution electrical sensor combining accelerometers and piezoelectric elements. We evaluate both sensor types on two tasks: texture classification and coefficient of dynamic friction prediction. Various configurations and resolutions were explored, along with multiple machine learning classifiers to determine optimal performance. The optical sensor achieved 99.9% accuracy on a challenging texture dataset, significantly outperforming the electrical sensor, which reached 82%. However, for dynamic friction prediction, both sensors performed comparably, with only a $\tilde{5}$% accuracy difference. We also found that the optical sensor retained high classification accuracy even when image resolution was reduced to 25% of its original size, suggesting that ultra-high resolution is not essential. In conclusion, the optical sensor is the better choice when high accuracy is required. However, for low-cost or computationally efficient systems, the electrical sensor provides a practical alternative with competitive performance in some tasks.

## 1. Introduction

Tactile sensing is an essential capability for both animals and robots, enabling tasks such as object manipulation, navigation, texture classification, and force estimation [1,2,3]. In nature there are numerous forms of tactile sensing. For instance, some nocturnal animals, such as whip spiders, utilise specialised low-resolution tactile sensors—antennae or antenniform legs—to effectively scan their environments so as to navigate without relying on vision [4]. Other species, including insects and small mammals, employ hairs to detect vibrations, which assist in navigation and surface manipulation and recognition [5]. In contrast, humans primarily rely on high-resolution tactile sensing via a very large number of receptors distributed throughout the skin, enabling sophisticated object manipulation and texture classification [6]. This diversity in tactile sensing mechanisms in nature is mirrored in the field of robotics, where tactile sensing is divided into four main categories: thermal, inductive, capacitive/electrical, and optical [7,8]. However, unlike in nature where we assume that natural selection has favoured the most appropriate sensor given constraints on brain size, energy, and the demands of the tasks, to understand the relative strengths and weaknesses of different sensor types for a robotic task we must compare them. To this end, this paper presents a comparison of sensors for tactile texture classification and friction prediction, focusing on optical and capacitive/electrical sensors as they are the most prominent approaches [3,9,10,11,12].

In recent years, optical tactile sensors, such as TacTips, have gained significant attention for their high-resolution, contact-based sensing capabilities [1]. These sensors utilise camera images to track the deformation of optical markers embedded in soft materials such as silicone gels, translating mechanical interactions into changes in visual features that can be processed using computer vision techniques for a variety of applications, including texture classification [13,14]. In contrast, electrical sensors typically have a relatively small number of input channels—often one for piezoelectric sensors and three for accelerometers. Such low-resolution electrical sensors—which have been shown to be highly versatile [2,15]—tend to require less processing and be much more cost-effective than optical tactile sensors, where the primary expenses are cameras and manufacturing costs. Thus, these two sensor types have contrasting characteristics. However, to date, there has been no extensive comparison of optical tactile sensors and low-resolution electrical tactile sensors on the texture classification problem. Although comparisons exist between various optical sensors [1] and between different electrical sensors [2], a rigorous comparison between capacitive/electrical and optical sensors is missing. Even in comprehensive papers such as [2], where the physical properties and typical energy consumption of different types of sensors are defined, the dataset and the task used to measure accuracy differed between sensor types. Inevitably, reviews of multiple studies analyse one type of sensor separately from another, using different datasets, making direct comparisons challenging [16]. This paper provides a detailed comparison of these two sensor types on the texture classification task.

Another related question, which has also been neglected until now, concerns the resolution of the optical sensor when used for the texture classification task. It is usually assumed that the highest resolution that the sensor is capable of should be used. There has been no examination of whether it is possible to scale down the image resolution without having any significant impact on the classification accuracy. Comparison of optical and electrical sensors across varying resolutions is essential to establish when and if optical sensing provides a tangible advantage. This question is directly addressed in this paper.

Within the field of tactile sensing, although texture classification has been established as a benchmark task, a standardised set of textures for comparison remains elusive. Much existing literature focuses on optimising individual tactile sensors to perform well on tactile datasets [10,11,12,17], which can vary greatly between different studies. These datasets often include diverse textures selected for convenience rather than consistency. In the research reported in this paper, we therefore created a new set of textures based on commonalities between previous datasets in order to improve comparability.

While rigorous comparative studies between optical and electrical tactile sensors on texture classification are lacking, there has been extensive individual testing of various sensor types. Accelerometers, for example, have been shown to classify multiple textures quite well with varying accelerations applied to the sensor [18]. Comparisons between accelerometers and piezoelectric sensors have been made, with arguments that accelerometers may be more effective for certain texture classification tasks [19]. However, these studies are often specific to particular applications; for instance, piezoelectric sensors [20] and capacitive sensors [21] have been explored for texture classification in dynamic environments, often yielding varying degrees of accuracy depending on the surface interaction, as well as the area of surface being tested.

Our main interest in tactile sensors is in relation to robot locomotion. Such applications demand sensors capable of adapting to continuous surface contact rather than discrete insertions (such as inserting a sensor into a fruit for classification [19]). Hence our sensors are dragged across the surface to be classified, mimicking the way an animal might stroke a surface with its foot to detect ground texture. This is also consistent with the most commonly used technique for artificial finger tactile texture classification [9].

This paper aims to fill the gaps identified above by rigorously comparing and exploring both optical (modified TacTips at varying resolutions) and electrical (piezoelectric and accelerometer-based) tactile sensing methods, using a dataset designed to encompass a diverse range of textures. In keeping with previous studies [9], we also assessed these sensors on a coefficient of friction identification task to evaluate their performance in broader applications of relevance to robotic locomotion. In addition, we evaluated the physical resolution of both types of sensing. Our findings indicate that while both methods demonstrate strong performance, optical sensors significantly outperform their electrical counterparts overall.

## 2. Methods and Materials

### 2.1. TacTip Construction

**Figure 1:**
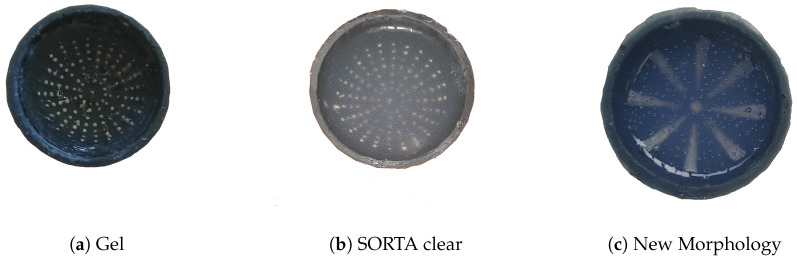
The different soft bodies for the skin of the TacTip. (**a**) The RTV27905 Gel in the original TacTip; the cloudier adaptations in (**b**,**c**) use the cheaper clear silicone, with (**c**) being the lower resolution design.

In the original TacTip design [22,23], RTV27905 silicone gel (Figure 1a) was used. However, we found that it can break easily—not a welcome property for a robot’s feet, which may be subject to considerable wear and tear. Therefore we selected SORTA-CLEAR silicone gel (in Figure 1b,c) as, although it is not as soft and sensitive as RTV27905 gel, it is significantly more robust.

To remove bubbles we placed the clear silicone in a vacuum tube and left it there for 10 min under contact force. The silicone was then poured into the TacTip skin over the painted markers. This assembly was placed in a small resin oven at 50 °C for two hours and left overnight to set. The resulting soft silicone tip was then glued onto the end of the TacTip 3D-printed casing. Internally there is a ring of LEDs, and at the opposite end a 2MP 50 fps USB Arducam webcam with a fisheye lens is mounted. The diameter of the TacTip is 42 mm, with an outer skin thickness of 2 mm. More detailed information on the sensor manufacture, and its general properties, can be found in the Supplementary Material.

A second TacTip version was created with fewer, larger optical markers in a different layout to investigate the previously unexplored impact of reduced marker resolution on performance. The marker layout was similar to that in the original but considerably less dense. Specifically, we were interested in whether or not fewer, larger markers—which thus produced lower resolution visual information on sensor deformations—were capable of producing high performance on the texture classification task. This question is of relevance because the new morphology (Figure 1c) is simpler and cheaper to manufacture than the original morphology, with its smaller optical markers, which requires more expensive equipment, such as high-resolution printers. The optical sensor read frequency was 10 Hz for both TacTip versions.

The total component costs of our TacTip sensor was approximately USD 100. However, cheaper 2MP cameras have now become available and could be used, which would reduce the cost to about USD 40–50. The setup costs for the standard TacTip will always be greater than for the new morphology version as more expensive filament is needed for high-resolution 3D printers. Hence, making TacTips with larger optical markers and cheaper 2MP cameras would be the lowest cost approach to making this kind of sensor.

### 2.2. PressTip Construction

**Figure 2:**
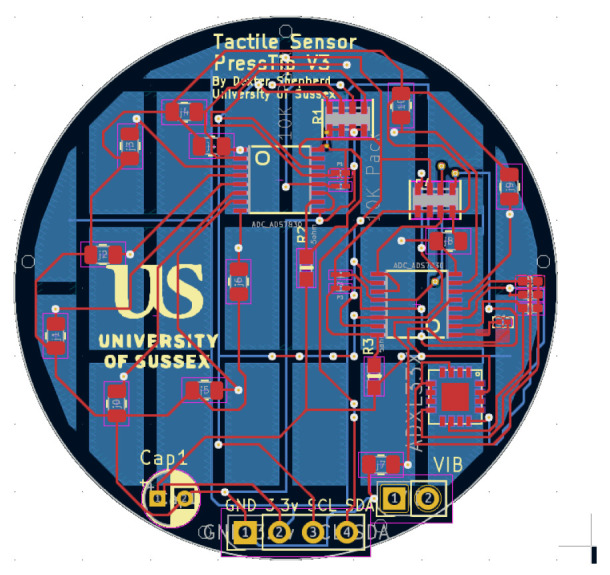
CAD design for the PressTip PCB. The tactile pads are shown in blue, and the top layer is shown in red. The main electronics such as the i2c bus ADC multiplexers, vibration sensors, and resistors are located in the top layer. The contact with the surface occurs underneath the sensor.

**Figure 3:**
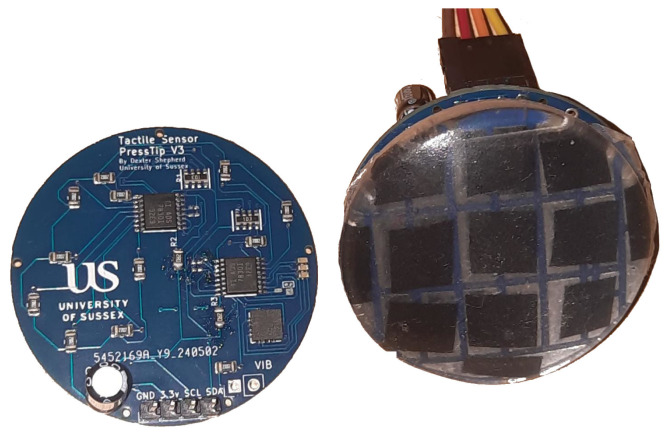
PCB showing both layers of the PressTip design. The sensor diameter is 42 mm, and each tactile pad covers a surface area of 10 × 10 mm^2^.

The PressTip is a novel sensor we developed to compare and combine multiple aspects of electrical tactile sensing. By using different combinations of the PressTip’s sensor, we can effectively provide different kinds of electrical sensors in our comparative study. The PressTip has a vibration-sensitive piezoelectric sensor (MEAS Flexible PVDF Piezo Polymer Film), 3-axis accelerometer (ADXl335), and an array of force sensors on the bottom side of the PCB. The force sensors were constructed using a conductive material (velostat), which changes resistance based on force; these are the black squares seen in Figure 2 and Figure 3. We previously showed that these force sensors are useful for a range of classification tasks [15], including under-foot edge detection. However, after performing preliminary experiments, we discovered that the information from the force sensor array was not suited to the texture classification task addressed in this paper. Hence, only vibration sensing from the piezoelectric sensor and the accelerometer were used in the work described here. Thus three modes of electrical sensing were used in the comparative studies: accelerometers only, piezoelectric only, and accelerometers plus piezoelectric.

The hybrid sensor makes use of 27 Ω resistors between each analogue reading input. The input voltage used is 3.3 V. The capacitors between the analogue read and accelerometer are 10 nF. The sensor has an average read frequency of 10.2 Hz. More detailed information on sensor construction can be found in the Supplementary Material.

These sensors were much cheaper to manufacture than the TacTip, costing around USD 4 each as PCBs and another USD 6 for silicone pads, velostat, and a piezoelectric sensor to be soldered, a total of USD 10.

### 2.3. Data Gathering and Dataset

**Figure 4:**
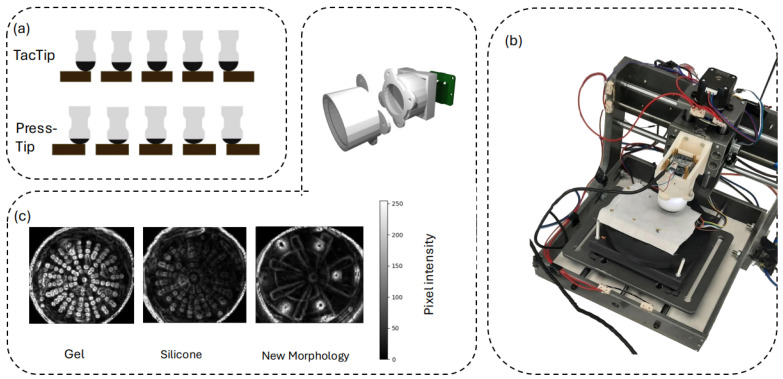
(**a**) The TacTip and PressTip sensors were lowered to the point of touch and dragged across the surface. (**b**) The rig with the TacTip attached to the z-axis. On the rig table is the cotton texture that has been bolted down. Further specifications can be found in the Supplementary Material. (**c**) Appearance of the optical sensor, different fillings, and designs after the Sobel filter is applied. The gel is much clearer, but the markers are still visible with the silicone.

A 3-degrees-of-freedom rig (x-axis, y-axis, z-axis), of dimensions 300 mm by 300 mm by 150 mm, was constructed to move a sensor around a 3D environment. This rig was used to gather texture data by stroking the sensor along 1.5 mm straight lines in 100 different directions along evenly spaced radii of a semicircle centred on the original touching point. The sensor moved across fixed-down samples from the texture set, as shown in Figure 4b. This is the most common way of gathering textural datasets; however, we additionally gathered a non-linear stroke dataset, which is discussed in Section 3.5.

**Figure 5:**
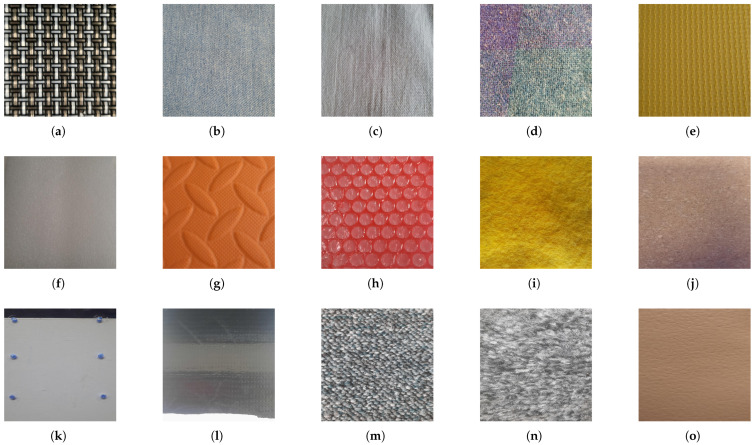
The 15 textures used in this study are shown in the figure; they were as follows: (**a**)—Interlaced mat. (**b**)—Denim Jeans. (**c**)—Cotton. (**d**)—Wool. (**e**)—Foam with small grooves (which we will refer to as Efoam). (**f**)—Foam with a smooth surface (which we will refer to as Ffoam). (**g**)—Foam with large grooves (which we will refer to as Gfoam). (**h**)—Bubble wrap. (**i**)—Felt. (**j**)—Cork. (**k**)—Flat Plastic. (**l**)—Concentrated rubber. (**m**)—Short carpet. (**n**)—Long carpet. (**o**)—Leather. The measured coefficients of dynamic friction for these textures are shown in Table 1.

Textural classification is often best approached as a temporal problem [24]. Recording data throughout the stroking motion captured the temporal aspects of the task. Recordings were gathered at various contact forces, using touching point forces of 0.0785–0.0824 N, 1.9031–1.9228 N, 3.0039–3.0235 N, and 4.336–4.367 N. The dataset consists of 3000 items gathered over 15 textures (pictured in Figure 5). Each dataset item contains multiple sensor readings concatenated over the time that the sensor is in contact with the surface (approximately 10 s).

Perhaps understandably, most papers on tactile sensing for texture classification have used textured materials that are easily accessible [10,11,12], and to date no standard texture set has been created. Past texture sets are often of different sizes, which hampers comparability because, as shown later in this paper, some texture classifiers perform well when distinguishing between small numbers of textures but degrade sharply as the number of classes increase. Established current datasets tend to use items that appear in the labs of the researchers. Often these include some form of carpet, hard materials, and fabrics. Our texture dataset [25,26] was designed to try and include commonalities between previous sets and to represent a range of material properties that a walking robot may come into contact with, such as indoor or flat outdoor surfaces (see Figure 5). These included soft/hard bodies, coarse/smooth surfaces, and surfaces with raised aspects.

Unlike many papers, we measured the dynamic coefficient of friction for each surface against a rubber tape, both as additional defining information for the dataset and to allow for more general applications (see Table 1). This tape does not match the textural properties of silicone tips exactly, but it does provide a good heuristic for surface roughness. The classification of the coefficient of friction can be very useful for gait control; for instance, in enabling slower careful movements on a slippery surface or faster, forceful movements on a surface with high grip.

### 2.4. Classification

#### 2.4.1. Texture Classification

As with much previous work in this area [9,10], texture classification was achieved by feeding readings from the tactile sensor into (pre-trained) machine-learning-based classifiers. The efficacy of four widely used machine learning classifier techniques were tested to determine which was most suited to the texture classification task for the TacTip and the PressTip. These were a random forest classifier (RFC) [27], which is an ensemble decision-tree method; a support vector machine (SVM) [28], which is a statistical learning method; a convolutional neural network (CNN) [29]; and a long short-term memory neural network (LSTM) [30]. These methods were chosen because they can all handle complex, noisy, high-dimension data. For the low-resolution electrical sensors, RFC, an SVM, LSTM, and a feedforward artificial neural network (ANN) were investigated. (The CNN was not suited to the much lower resolution of the data).

For the optical sensor (TacTip) dataset [25], the camera image was flattened into a vector of size *h* × *w*, where *h* is the height and *w* the width of the image. In order to incorporate the important temporal element discussed earlier into the classifier input, the image vectors over *T* read cycles (frames) were concatenated, giving a classifier input vector of size *h* × *w* × *T*. Similarly, for the electrical sensor dataset, concatenated classifier input vectors of size *s* × *T* were used, where *s* is the number of electrical sensors. In preliminary experiments, a systematic search of *T* values ranging from 1 to 20 was performed. A value of 10 was generally found to be good, but for CNN classifiers for the TacTip, a value as low as 4 still provided high accuracy.

After preliminary experiments with the TacTip, we found that for the CNN classifier, a hidden layer of 128 nodes, along with 15 output nodes (one for each possible texture class), was sufficient to achieve high accuracy. For the LSTM, a hidden layer of 50 units, with 15 output nodes, was optimal. See Table 2 for details of other classifier parameters used; they were all determined from preliminary experiments. After training for only 100 epochs, the neural classifiers gave very high accuracy with the TacTip.

The LSTM classifiers for the PressTip electrical sensors made use of the same parameters as for the optical data (except for the number of inputs). These classifiers took longer to train on the electrical dataset to find maximum accuracy—up to 60,000 epochs. An investigation of hidden layer sizes for the PressTip revealed that accuracy drops with less than 25–30 hidden nodes, and loss minimizes quicker with 40–50 nodes. Hence we also used 50 nodes in the hidden layer for the PressTip to allow quicker convergence. The feedforward ANNs levelled out at a lower accuracy during training, though with fewer epochs (1000). All classifiers were trained on data from all 15 textures.

#### 2.4.2. Friction Classification

For friction detection, we employed regression models (random forest regression [27]) to capture complex relationships between the temporal data and friction acting on the sensor. We used a range of regression models (Linear [31], Ridge [32], Logistic [33]) to begin with but discovered in preliminary experiments that the random forest classifier significantly outperformed the other regression models, as it did the neural classifiers—see the Supplementary Material for further details. The random forest regression model used 100 estimators, a maximum depth of 25 (there was no benefit in going above this), and the squared error criterion. The initial random state was set to zero.

#### 2.4.3. Data Preprocessing

The optical data was converted to greyscale and passed through a Sobel filter [34] using a kernel of 3 × 3. In later experiments the optical image was re-scaled using area interpolation. This allowed us to investigate the role of resolution in the optical sensors. In these experiments the image was resized to to the following percentages of the original size: 5%, 10%, 15%, 20%, 25%, 30%, 40%, 50%, 60%, 70%, 80%, 90%, and the original size. Data from the PressTip (piezoelectric and accelerometer) was preprocessed using a Butterworth filter to reduce noise and remove spikes. The Butterworth filter was chosen after preliminary experiments with many types of filter to ascertain which gave the best accuracy (see Supplementary Material for details of these experiments).

All data was scaled using min-max scaling to convert values into the range 0–1. 80% of the dataset (2400 samples of *T* frames) was used for training, and 20% (600 samples) was used for testing. See the Supplementary Material for further hyperparameter experiments and detailed justification of the methods used.

## 3. Results

### 3.1. Textural Classification

The results of the comparative texture classification experiments are shown in Table 3 for the various sensor–classifier combinations. It is clear from the table that the optical sensors have a much higher accuracy across all classifier types compared to the electrical sensors. This is confirmed by the statistical analysis shown in Table 4. However, the electrical sensor combining the accelerometers and piezoelectric sensor offers good accuracy when using a LSTM classifier or a RFC classifier. The TacTip employing the new marker morphology performs slightly worse than with the original marker morphology but is still very accurate. Overall, the best performing sensor is the standard-silicone-filled TacTip, particularly in combination with CNN or LSTM classifiers. While SVM classifiers performed well, they took much longer to train than the other methods.

**Figure 6:**
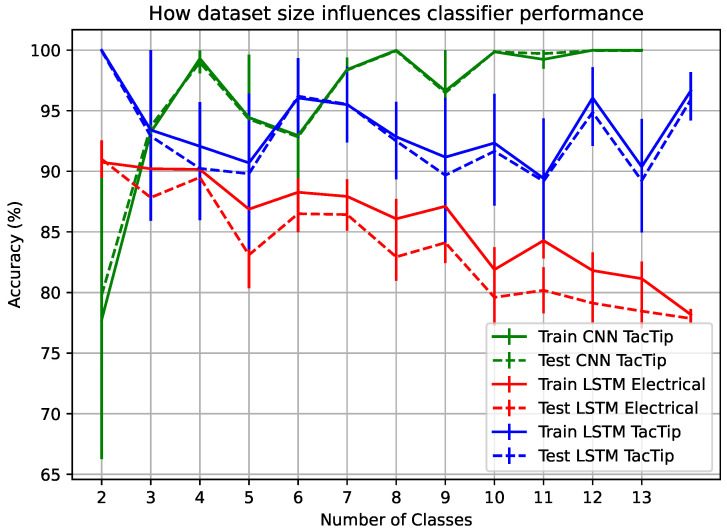
The influence of the number of texture classes in the dataset on classifier accuracy. It is worth noting that it took significantly fewer epochs of training for the smaller number of classes with the electrical classifiers. The results show an average across 5 trials.

The number of classes in a dataset can impact classification accuracy. A binary classification between two textures is much simpler than accurately distinguishing between thirty textures. Hence we investigated classification performance in relation to the number of texture classes. Because some textures might be easier to distinguish than others, we measured average performance across five trials for each number of classes in the dataset, with a random set of classes chosen on each trial. By evaluating the trend, we can estimate how robust the sensors are to increasing dataset sizes. The results are shown in Figure 6. In this set of experiments, the best optical and electrical sensor–classifier combinations from the main comparative experiments (see Table 3) were used. As seen in Figure 6, only the electrical PressTip accuracy reduces as the number of classes increases, suggesting that its low resolution makes it difficult to find relationships between larger numbers of classes.

The statistical analysis shown in Table 4 confirms that optical sensors outperform electrical sensors in texture classification. Note that there was no significant difference in the performance of the two best classifiers, CNNs and LSTM, for the TacTip. Additionally, the original TacTip design significantly outperforms the newer morphology, suggesting that a higher resolution of temporal movements is important for this task.

### 3.2. Friction Coefficient Detection

**Figure 7:**
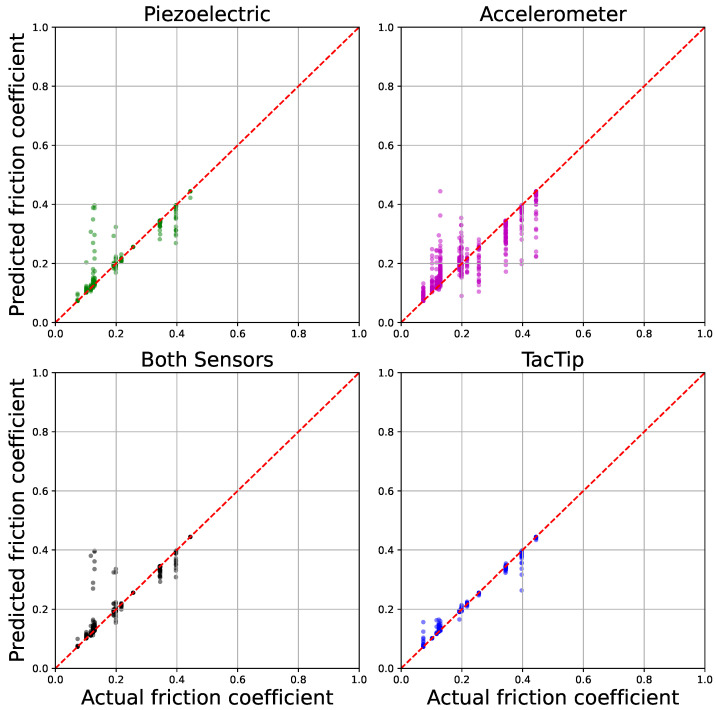
Friction prediction lines of best fit from the regression models for unseen test data across the various sensors.

Friction detection model performance was calculated using the mean squared error (MSE) between predicted and true values with the mean calculated over all textures in the dataset. Although there is significant noise in the voltage readings of the electrical sensors, the random forest regression model dealt well with this. Table 5 displays the results of the coefficient of friction detection experiments, showing a smaller error from the optical sensor. However, the relative performance of the electrical sensors is good and better than for the texture classification task. Figure 7 shows lines of best fit on a graph plotting the actual friction against the predicted friction from our regression models. The TacTip values are more closely clustered around the line of best fit. Although the electrical sensor readings are noisier and wider spread, their regression model produced a close match to the actual values. The results shown in Table 5 are for the clear-silicone TacTip, and for various configurations of the electrical sensor (which also has a silicone tip).

### 3.3. Texture Classification Contact Force Generalisation

In order to test the influence of the applied contact force to texture classification, we gathered data for our texture set at contact forces unseen during classifier training. We tested the best trained classifiers from the original texture classification task on these unseen contact forces. The results for the optical TacTip and electrical PressTip (piezoelectric plus accelerometers) sensors are shown in the top and bottom parts of Table 6, respectively. Though the main dataset was gathered on the three contact force ranges 0.0785–0.0824 N, 1.9031–1.9228 N, and 3.0039–3.0235 N, for the generalisation experiments we removed the contact force that was being tested from the dataset. For example, to test 0.0785–0.0824 N as the unseen contact force (first and fifth rows of Table 6), the classifiers were tested at this contact force having been trained on 1.9031–1.9228 N and 3.0039–3.0235 N only. To evaluate robustness, we selected the top-performing neural classifier and the best-performing non-neural method for each sensor. The tables demonstrate the robustness of the optical classifiers, highlighting their ability to generalize to unseen contact force conditions. The electrical sensor struggled to generalise to unseen contact forces, likely due to movement-related noise—slower movements produced different readings. In contrast, the optical sensor’s high dimensionality retained useful information despite noise.

**Figure 8:**
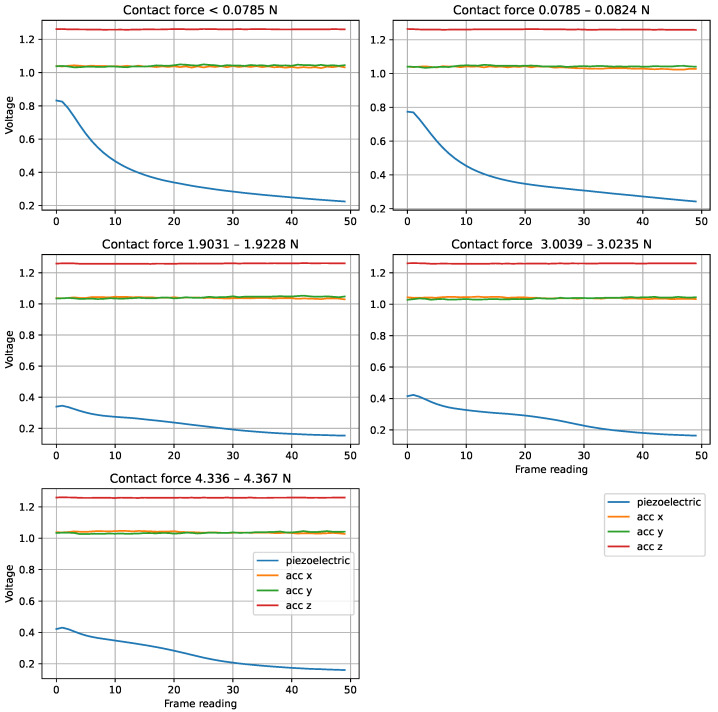
Various contact forces applied on the PressTip sensor as it was stroked across the Plastic surface only. We averaged 200 trials in various directions to elucidate any trends with readings and contact force.

In order to investigate the issues faced by the electrical sensors in generalising to unseen contact forces, further experiments and analysis were carried out. When we averaged the sensor readings across trials at various contact forces using the PressTip, we observed a clear trend as contact force increased. Figure 8 shows these averaged readings. One of the most noticeable changes is the initial value of the piezoelectric signal, which tends to decrease as contact force increases. This is likely due to reduced vibration at higher contact forces, where the sensor is more firmly in contact with the surface. The initial movement often reflects the sensor overcoming static friction. Another notable change is the smoothness of the accelerometer signal. Analysis of the variance in the averaged accelerometer readings across 200 trials showed a decrease in variance as contact force increases, indicating more consistent readings under higher loads.

The very low resolution and noisy nature of the electrical sensor makes the classifier learning task significantly harder, which helps to explain why the PressTip classifiers took longer to train than the TacTip classifiers. While the PressTip classifiers were able to extract features from the data to give good performance in conditions similar to those during training, they were not able to generalise to unseen contact forces because the properties of the sensor signals are partly determined by contact force, as indicated by the analysis shown in Figure 8.

### 3.4. Effect of Resolution on TacTip

**Figure 9:**
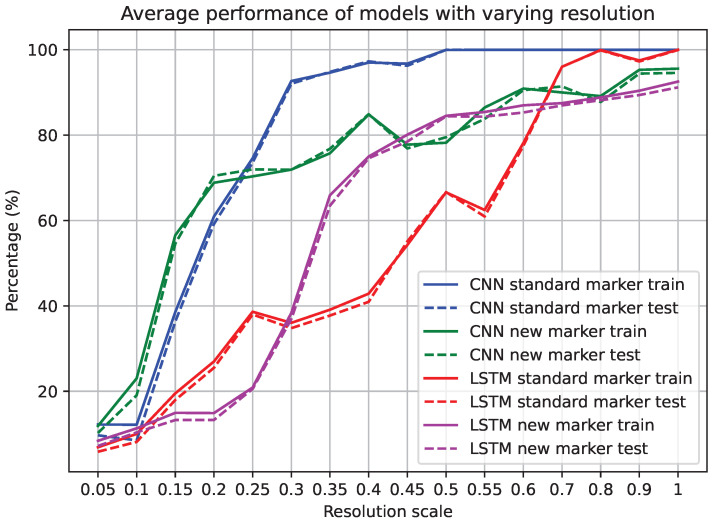
Accuracies of best TacTip classifiers (from the main texture classification experiment) when trained on data at different spatial resolutions. The results are averaged over 5 trials per resolution.

Texture classification experiments were repeated for the TacTip over a number of different image resolutions. At each resolution the classifiers were trained and then tested on the unseen data. The results shown in Figure 9 reveal that higher resolution generally leads to higher accuracy. However, we discovered that for CNN classifiers with the original marker morphology, the image resolution could be reduced to 25–30% while maintaining accuracy above 90%. At 50%, resolution accuracy was above 99%. Other classifiers did not perform as well as this, but most had high accuracy at significantly reduced image resolution. This experiment shows that high resolution is unnecessary for optical tactile sensing for texture classification.

### 3.5. Non-Linearity Study

In the experiments discussed so far, in keeping with all previous work on texture classification, the sensors performed linear stroking movements (as described in Section 2). While very good texture classification was demonstrated with this kind of movement, in applications such as robotics, non-linear movements often occur. Hence we investigated how the sensor–classifier pairs performed on sensations they were not trained on, induced by non-linear movements. To do this, we collected a non-linear movement dataset by moving the sensor in circular movements of increasing radii (1 cm, 1.75 cm, and 2.5 cm) while increasing the contact force by approximately 0.167 newtons on each iteration. This dataset was preprocessed using the same techniques as employed for the main experiments, as described earlier in Section 2. The sensor–classifier pairs trained on the linear movement dataset, as detailed in Section 3, did not generalise to non-linear motion. While the main focus of this paper is on developing a model that can classify texture, in order to better understand the potential for a wider range of applications, including in robotics, further investigations were carried out into how to deal with non-linear movement, as detailed below.

One approach to allowing generalisation to non-linear movement is to train classifiers on enough variations in direction and contact force that they become robust to non-linear movement. We investigated this approach by training classifiers for texture classification on a hybrid dataset comprising the non-linear dataset described above and the original linear dataset described in Section 2 for both the TacTip and the PressTip. The approach was successful, achieving results comparable with those for the linear dataset results as described in Section 3.1. Classifiers were able to generalise over both unseen linear and non-linear movement; see Table 7 below for the results.

Future work could involve incorporating SO(3) rotations to make the model invariant to orientation, thereby improving robustness to unseen sensory inputs, or converting the images to a latent space using an autoencoder to train models on a more compressed feature space. Making a fully generalisable model is a significant paper in its own right and is outside the scope of the research described here. However, the preliminary results shown in Table 7 are very promising.

## 4. Conclusions

Across experiments, the optical sensor consistently outperformed the electrical sensors, likely due to its higher spatial resolution, which provides richer detail for classifier training. In contrast, the lower resolution of the electrical sensors made it more susceptible to noise. However, the electrical sensor was able to classify texture at a good accuracy (>80%), and when predicting friction, the performance gap between the two sensors was reduced to just 5%, suggesting that the electrical sensors can still offer competitive accuracy in this context. Given the significantly lower cost of electrical sensors, users must weigh the trade-off between cost and accuracy. Additionally, the lower data and processing requirements of electrical sensors make them more suitable for some embedded robotic applications, where memory constraints can make optical sensors less practical due to the higher data storage and processing needs associated with their cameras. Future work will include further studies of the role of TacTip optical marker resolution, and an investigation into whether or not performance on the tasks described in this paper is affected by marker patterns. An investigation into the reduction in image size while using larger markers could be an interesting avenue. In addition, the fusion of electrical and optical components into a hybrid sensor would provide richer sensory information, which might further improve robustness to non-linear data. This is a potentially fruitful direction for future investigations of texture and friction classification in robotic applications involving complex sensor movements. Other kinds of sensor fusion, including the incorporation of barometric-style pressure sensors [35], may also be useful in future wider robotic applications. If memory or cost is not an issue, then optical sensors have a higher accuracy and will perform more robustly. However, this study has shown that optical tactile sensors can operate at significantly reduced image resolution without losing accuracy. This reduces processing requirements, which might be a consideration in some applications.

## Figures and Tables

**Table 1:** Friction coefficients for each material in Figure 5.

Texture	Friction	Texture	Friction
FFoam	0.396798	Cork	0.344905
Flat	0.343766	Long Carpet	0.128852
Plastic	0.101801	Short Carpet	0.197868
Leather	0.255904	GFoam	0.191812
Felt	0.116275	EFoam	0.073127
Wool	0.198984	LacedMatt	0.124083
Bubble	0.217116	Cotton	0.129354

**Table 2:** The parameters of the classifiers used across experiments, as determined by preliminary investigations. The total input size for the CNN, RFC, and SVM classifiers includes concatenated data over *T* sensor cycles as explained in the main text. The electrical sensor data format is separated by commas showing the piezoelectric sensor, accelerometer, and both.

Parameter	Description
**LSTM**	
Optical input image size	110 × 120 = 13,200
Num electrical sensors	1, 3, 4
Num nodes (hidden, output)	(50, 15)
Sequence length (T)	optical = 4; electrical = 10
Activation	none
Learning rate	0.005
Loss function	Cross Entropy Loss
Optimizer	Stochastic Gradient Descent
**CNN**	
Optical input image size	13,200
Num nodes (hidden, output)	(128, 15)
Kernel size	3
Stride	1
Pooling type	Max
Activation	ReLU
Learning rate	0.005
Loss function	Cross Entropy Loss
Optimizer	Stochastic Gradient Descent
Sequence length (T)	4
**RFC**	
N estimators	100
Criterion	Gini
Min depth	8
Random state	0
**SVM**	
C	1.0
Kernel	RBF
Decision function	OVO
**ANN**	
Num electrical sensors	1, 3, 4
Num nodes (hidden, output)	(128, 15)
Activation	ReLU
Learning rate	0.005
Sequence length (T)	10
Loss function	Cross Entropy Loss
Optimizer	Stochastic Gradient Descent

**Table 3:** Results of the comparative texture classification experiments using the TacTip (TT), with standard silicone tip (Sil), and the new morphology (NM), and the various PressTip (PT) configurations—piezoelectric denoted by P and accelerometer by A. The table shows the average ($\bar{X}$) accuracy for training and unseen test data across 20 trials, along with the best and standard deviation (std) of the results. The results for the optical sensors are for the full image resolution.

Sensor	Classifier	${\bar{X}}_{\mathrm{test}}$	${\bar{X}}_{\mathrm{train}}$	Std Test	Max Test
TT Sil	SVM	99.96%	100%	0.0005	100%
TT Sil	RFC	99.9%	99.98%	0.025	100%
TT Sil	CNN	99.97%	99.99%	0	99.99%
TT Sil	LSTM	98.1%	99.1%	0.018	99.9%
TT NM	CNN	89.25%	90.31%	2.7	94.2%
TT NM	LSTM	95.71%	96.28%	0.027	99.2%
PT P	SVM	70%	70.4%	0.01	71.6%
PT A	SVM	44.75%	51.1%	0.025	49.1%
PT A & P	SVM	55.45%	57.8%	0.03	63.8%
PT P	RFC	78.7%	99.96%	0.014	76.6%
PT A	RFC	62.31%	100%	0.02	65.6%
PT A & P	RFC	89.6%	100%	0.01	99.2%
PT P	ANN	66.5%	66.5%	0.768	67.5%
PT A	ANN	39.57%	43.94%	0.759	41%
PT A & P	ANN	65.59%	64.84%	1.101	66.5%
PT P	LSTM	77.37%	85.03%	0.67	78.6%
PT A	LSTM	38.53%	42.1%	2.6	42%
PT A & P	LSTM	85.5%	90%	0.67	86.5%

**Table 4:** Statistical analysis of texture classification relative performance of the best optical and electrical sensor–classifier combinations on unseen test data using pairwise Wilcoxon rank sum tests with Bonferroni correction for multiple comparisons. Optical1 denotes the use of the standard TacTip, Optical2 denotes the use of the new Morphology. A total of 20 trials on each sensor–classifier combination were used.

Model 1	Model 2	*p*_Value	Significant
Optical1 LSTM	Piezo & Acc LSTM	$6.14\times 10^{-8}$	Yes
Optical1 CNN	Piezo & Acc ANN	$6.30\times 10^{-8}$	Yes
Piezo & Acc LSTM	Piezo LSTM	$6.38\times 10^{-8}$	Yes
Optical1 CNN	Optical1 LSTM	0.02	No
Optical1 CNN	Optical2 CNN	$7.92\times 10^{-9}$	Yes

**Table 5:** Friction prediction results showing mean squared error (MSE) between actual values and random forest regression model (RFR) predictions on the test data. We refer to piezoelectric sensors as piez and accelerometers as acc. The results for the TacTip were all gathered using the original image size. We trialled each model 10 times.

Sensor	Regression Model	Min MSE	Train MSE Average
Piez	RFR	0.022	0.024
Acc	RFR	0.019	0.021
Acc & Piez	RFR	0.018	0.021
TacTip	RFR	0.0043	0.0049

**Table 6:** Texture classification accuracy at unseen contact forces for the silicone TacTip sensor (first 4 rows) and the PressTip sensor (following 3 rows). The results are averaged over 10 trials for each texture.

Force (Newtons)	Sensor	LSTM	SVM	RFC
0.0785–0.0824	TacTip	100%	100%	100%
1.9031–1.9228	TacTip	100%	100%	100%
3.0039–3.0235	TacTip	100%	100%	100%
4.336–4.367	TacTip	100%	100%	100%
0.0785–0.0824	PressTip	4%	6.4%	12.5%
3.0039–3.0235	PressTip	15%	10.9%	10 %
4.336–4.367	PressTip	16%	7.13%	11 %

**Table 7:** Comparison of PressTip and TACTIP texture classification performance trained on linear and non-linear data from 20 trials. The best performing classifier types from the previous linear dataset experiments were used.

Sensor	Classifier	Test Accuracy	Train Accuracy	Std
PressTip	RFC	77.3%	100%	0.00%
TACTIP	RFC	97.58%	98%	0.00%
PressTip	LSTM	57.8%	85.3%	0.011%
TACTIP	LSTM	83.71 %	84.9%	0.00%

## Supplementary Materials

The following supporting information can be downloaded at: https://www.mdpi.com/article/10.3390/s25164971/s1, File S1: document with extended details of experimental design and sensor development.

## References

[1] Lepora N.F., Lin Y., Money-Coomes B., Lloyd J. (2022). Digitac: A digit-tactip hybrid tactile sensor for comparing low-cost high-resolution robot touch. IEEE Robot. Autom. Lett..

[2] Meribout M., Takele N.A., Derege O., Rifiki N., Khalil M.E., Tiwari V., Zhong J. (2024). Tactile sensors: A review. Measurement.

[3] Kakani V., Cui X., Ma M., Kim H. (2021). Vision-based tactile sensor mechanism for the estimation of contact position and force distribution using deep learning. Sensors.

[4] Santer R.D., Hebets E.A. (2011). The sensory and behavioural biology of whip spiders (Arachnida, Amblypygi). Advances in Insect Physiology.

[5] Barth F.G. (2019). Mechanics to pre-process information for the fine tuning of mechanoreceptors. J. Comp. Physiol. A.

[6] Wei Y., Marshall A.G., McGlone F.P., Makdani A., Zhu Y., Yan L., Ren L., Wei G. (2024). Human tactile sensing and sensorimotor mechanism: From afferent tactile signals to efferent motor control. Nat. Commun..

[7] Pirozzi S. (2020). Tactile Sensors for Robotic Applications. Sensors.

[8] Lee M.H., Nicholls H.R. (1999). Tactile sensing for mechatronics—A state of the art survey. Mechatronics.

[9] Jamali N., Sammut C. Material classification by tactile sensing using surface textures. Proceedings of the 2010 IEEE International Conference on Robotics and Automation.

[10] Huang S., Wu H. (2021). Texture recognition based on perception data from a bionic tactile sensor. Sensors.

[11] Ward-Cherrier B., Pestell N., Lepora N.F. NeuroTac: A neuromorphic optical tactile sensor applied to texture recognition. Proceedings of the 2020 IEEE International Conference on Robotics and Automation (ICRA).

[12] Gupta A.K., Nakagawa-Silva A., Lepora N.F., Thakor N.V. (2021). Spatio-temporal encoding improves neuromorphic tactile texture classification. IEEE Sens. J..

[13] Ward-Cherrier B., Cramphorn L., Lepora N.F. (2016). Tactile manipulation with a TacThumb integrated on the Open-Hand M2 gripper. IEEE Robot. Autom. Lett..

[14] Stone E.A., Lepora N.F., Barton D.A.W. Walking on TacTip toes: A tactile sensing foot for walking robots. Proceedings of the 2020 IEEE/RSJ International Conference on Intelligent Robots and Systems (IROS).

[15] Shepherd D.R., Husbands P., Philippides A., Johnson C. Versatility of low-resolution tactile sensing for edge and pose detection. Proceedings of the 2024 5th International Conference on Artificial Intelligence, Robotics and Control (AIRC).

[16] Chen W., Khamis H., Birznieks I., Lepora N.F., Redmond S.J. (2018). Tactile sensors for friction estimation and incipient slip detection—Toward dexterous robotic manipulation: A review. IEEE Sens. J..

[17] Wang D., Teng Y., Peng J., Zhao J., Wang P. (2023). Deep-learning-based object classification of tactile robot hand for smart factory. Appl. Intell..

[18] Strese M., Lee J.-Y., Schuwerk C., Han Q., Kim H.-G., Steinbach E. A haptic texture database for tool-mediated texture recognition and classification. Proceedings of the 2014 IEEE International Symposium on Haptic, Audio and Visual Environments and Games (HAVE) Proceedings.

[19] Iwatani S., Akimoto H., Sakurai N. (2013). Acoustic vibration method for food texture evaluation using an accelerometer sensor. J. Food Eng..

[20] Guo Q., Al G.A., Martinez-Hernandez U. (2024). VibroTact: Soft piezo vibration fingertip sensor for recognition of texture roughness via robotic sliding exploratory procedures. IEEE Sens. Lett..

[21] Hughes D., Correll N. (2015). Texture recognition and localization in amorphous robotic skin. Bioinspir. Biomimetics.

[22] Chorley C., Melhuish C., Pipe T., Rossiter J. Development of a tactile sensor based on biologically inspired edge encoding. Proceedings of the 2009 International Conference on Advanced Robotics.

[23] Lepora N.F. (2021). Soft biomimetic optical tactile sensing with the TacTip: A review. IEEE Sens. J..

[24] Cao G., Zhou Y., Bollegala D., Luo S. Spatio-temporal attention model for tactile texture recognition. Proceedings of the 2020 IEEE/RSJ International Conference on Intelligent Robots and Systems (IROS).

[25] Shepherd D.R. (2024). Optical Tactile (TacTip) Dataset for Texture Classification.

[26] Shepherd D.R. (2024). Electrical Tactile Dataset (Piezoelectric and Accelerometer) for Textures.

[27] Kleinberg E.M. (1990). Stochastic discrimination. Ann. Math. Artif. Intell..

[28] Bennett K.P., Campbell C. (2000). Support vector machines: Hype or hallelujah?. ACM SIGKDD Explor. Newsl..

[29] LeCun Y., Bengio Y., Hinton G. (2015). Deep learning. Nature.

[30] Hochreiter S., Schmidhuber J. (1997). Long short-term memory. Neural Comput..

[31] Freedman D.A. (2009). Statistical Models: Theory and Practice.

[32] Hilt D.E., Seegrist D.W. (1977). Ridge, a Computer Program for Calculating Ridge Regression Estimates.

[33] Conklin J.D. (2002). Applied Logistic Regression.

[34] Sobel I., Feldman G. (1968). An isotropic 3 × 3 image gradient operator. Stanford AI Project (SAIL) Meeting.

[35] Galaiya V.R., Asfour M., de Oliveira T.E.A., Jiang X., da Fonseca V.P. (2023). Exploring tactile temporal features for object pose estimation during robotic manipulation. Sensors.

