# Supplementary material for: Texture and Friction Classification: Optical TacTip vs. Vibrational Piezoeletric and Accelerometer Tactile Sensors
Source: Sensors (Basel). 2025 Aug 11;25(16):4971. doi: 10.3390/s25164971 (PMC12390442; doi:10.3390/s25164971)
Supplement: Supplementary file 1 [file sensors-25-04971-s001.zip › sensors-3760215-supplementary.pdf]

Supplementary Material for: "Texture and Friction Classification: Optical TacTip vs. Vibrational Piezoeletric and Accelerometer Tactile Sensors"

Dexter R. Shepherd, Phil Husbands, Andrew Philippides and Chris Johnson
(corresponding author: d.r.shepherd@sussex.ac.uk)

1 Extended Details of Experimental Setup

1.1 Optical Sensor Development

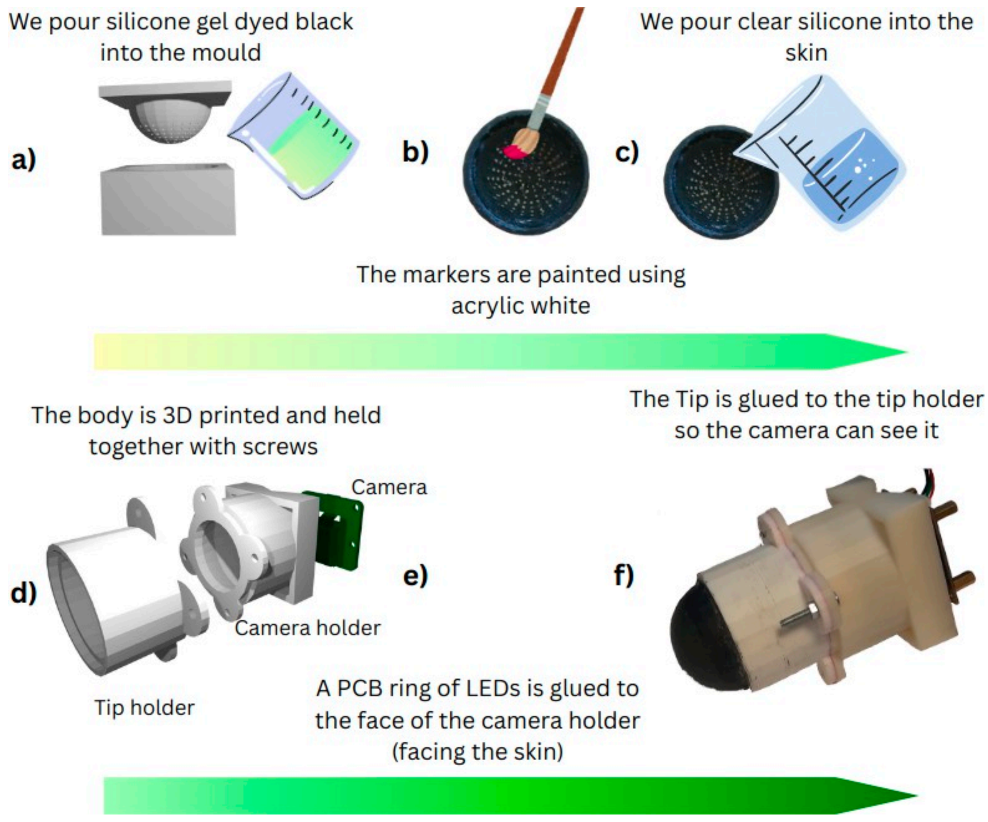

Figure S1: Process of manufacturing the TacTip

Figure S1 gives an overview of the way the TacTip sensor was manufactured. The TacTip parts were 3D printed and consisted of a camera mount, main body, tip and ring. The camera mount was edited on CAD software to mount our webcam (Arducam USB wide-angle lens), the main body was attached on to the mound via a series of screws. The files were printed using PLA filament on an industrial high-resolution 3D printer. Between the tip and the main body there is a PCB of small LEDs that provide light. The camera

detects reflection of the LED light off the white painted tips on the surface of the skin. The PCB made use of 6 surface-mount LEDS and 6 surface-mount capacitors.

The PCB was mounted in the rim of the main body, where the LEDs face the skin, to illuminate the skin for the camera. The wires are pressed into a printed groove that leads to a hole allowing for power connection.

The camera was mounted onto a custom 3D printed part that connected to a USB wide-angle lens webcam. The power of the LED ring was connected to the power of the USB camera for wiring efficiency.

The silicone skins were produced within a 3D-printed plastic mould. This mould was measured to fit the silicone inside a thin layer, with small holes in it to create the tips that would later be painted white to act as optical markers. The moulds were painted in high resolution to capture the 2mm diameter of the optical markers. The outcome was a 42mm diameter exterior and 38mm diameter interior, where the skin has a 2mm thickness.

Silicone was made and dyed black to prevent light interference or any more glare than the background levels. After being poured into the mould and left for twenty-four hours, a solid yet flexible TacTip was produced. The mould required a lubricant spray over the plastic layers to prevent the tips from getting pulled off. To paint the tips we used a thin layer of plastic sheet with acrylic paint. The tip was turned inside out (so the marker positions were on the exterior) and the paint gently dabbed over.

We modified the original TacTip design to make use of a clear silicone gel (SORTA-CLEAR hardness 12 shown in Figure S2b,c instead of the RTV27905 gel used in the original. RTV27905 (shown in Figure S2a) is softer and more sensitive, however, it breaks easily. Since a major wider motivation for this work is to develop tactile sensors suitable for a walking machine's feet, which may need to bear considerable weight, we switched to SORTA-CLEAR. When we placed too much weight on a RTV27905 gel filled TacTip it would break (as seen in Figure S2d). To remove bubbles we placed the clear silicone in a vacuum tube and left it there for 10 minutes under pressure. The silicone was then poured into the TacTip skin over the painted markers. This assembly was placed in a small resin oven at 50°C for two hours and left overnight to set.

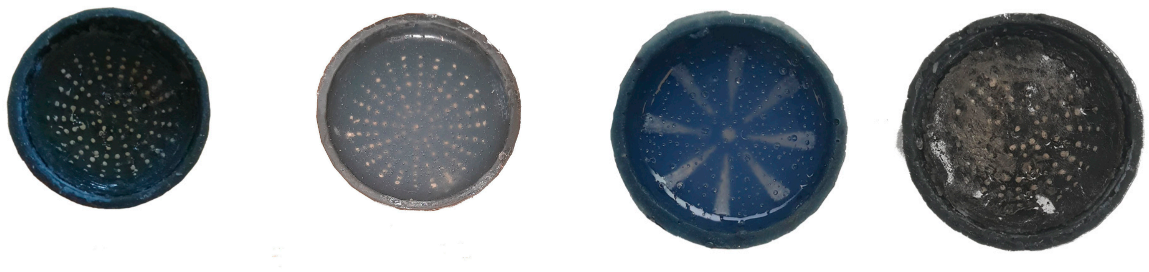

(a) Gel (b) SORTA clear (c) New Morphology (d) Broken Gel

Figure S2

The resulting soft silicone tip was then glued onto the end of the TacTip 3D printed casing. Internally there is a ring of LEDs, and at the opposite end a 2MP 50fps USB Arducam webcam with fisheye lens is mounted. The diameter of the TacTip is 42mm, with an outer skin thickness of 2mm.

A second version of the TacTip was also created, for comparison, by modifying the skin to use fewer and larger optical markers laid out with a different morphology. The new morphology (Figure S2c) is simpler and cheaper to manufacture than the original morphology, with its smaller optical markers, which requires more expensive equipment, such as high resolution printers. The optical sensor read frequency was 10Hz.

1.2 Electrical Sensor Development

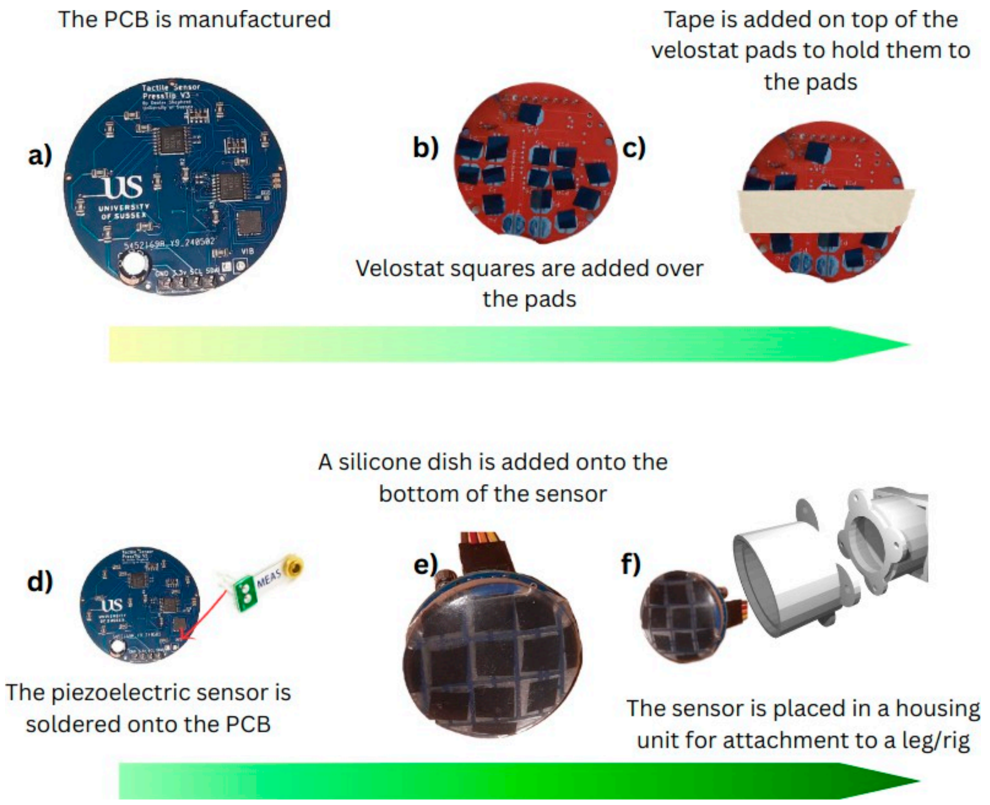

Figure S3: Process of manufacturing the PressTip sensor, after the PCB is ordered.

We initially experimented with force-sensitive resistors embedded in a silicone body to create low-resolution skin sensors with soft compliance. However, literature indicated that vibration sensing is the standard approach for texture classification. Several types of vibration sensors were considered.

The Low-Cost Ceramic (LCC) vibration switch sensor operates on the principle of inertia. It contains a conductive mass—typically a small metal ball or a spring-mounted element—that contacts electrical terminals when stationary. This sensor showed promise in early prototypes, performing well as a binary classifier between rough and smooth terrain. However, it proved too noisy for our application, leading us to explore piezoelectric sensors and accelerometers instead.

Piezoelectric sensors detect vibrations and mechanical strain using the piezoresistive effect, where a material's electrical resistance changes under mechanical stress. These sensors typically feature a piezoresistive material (often silicon) bonded to a flexible substrate. Vibrations deform the material, altering its resistance, which is then converted into a proportional electrical signal.

Accelerometers measure vibration by detecting changes in acceleration. They contain a mass suspended by a spring inside a casing. Vibration causes the mass to move, deforming the spring and generating an electrical signal proportional to the acceleration. Due to their compact size and high sensitivity, accelerometers are widely used across electronics, machinery, and vehicles.

These insights led to the development of the PressTip (Figure S3), which integrates both a piezoelectric sensor and an accelerometer on a single board. Originally intended to allow side-by-side comparison of both sensor types on the same texture, we found that using both simultaneously improved classification accuracy—an outcome detailed in the main paper.

1.3 Test Rig

The data collection rig had the parameters listed in Table S1. We gathered three sub datasets within the main dataset. Linear motion data was collected from straight line movements in varying directions. Non-linear data was collected from circular movements at various radius values (see Section 2 of this document). Then finally data with varying pressure from sensor movements across the rig table.

Parameter	Description
**Rig and Motor Parameters**	
Axis Number	X, Y, Z (labels for each axis)
Motor Type	ANFOD Stepmotor 17HD4401S-23B (43N)
Step Resolution	1.8°
**Sensor Parameters**	
External Silicone	15A
Internal Silicone	12A
Electrical Silicone	40A
**Experimental Parameters**	
Scan Resolution	1 ± 0.5 (mm)
Contact Force	308–588 (g)

Table S1: Rig and Experimental Parameters

1.4 Data preprocessing selection

1.4.1 TacTip

We used a number of optical preprocessing techniques to determine the structural importance, feature importance and noise influence. We used a vast range of preprocessing techniques (28 techniques) outlined in Table S2 to experiment with a spectrum of values across the three dimensions we investigated (edge, noise and contrast), in order to determine the best preprocessing methods to use for the main experiments described in the main paper.

**Technique**	**Parameters**
Sobel Filter	kernel size = 3
Canny Edge Detector	threshold1 = 0, threshold2 = 20
Laplacian Filter	-
Prewitt Filter	Custom kernels
Scharr Filter	-
Bilateral Filter	d = 9, sigmaColor = 75, sigmaSpace = 75
Gaussian Noise Addition	mean = 0, std = 25
Salt and Pepper Noise	prob = 0.02
Speckle Noise	std = 0.1
Poisson Noise	-
Median Filtering	kernel size = 5
Gaussian Blur	kernel size = 5, sigma = 1
Wavelet Denoising	db1 wavelet, level = 1
Histogram Equalization	-
Adaptive Histogram Equalization	clipLimit = 2.0, tileGridSize = (8,8)
Gamma Correction	gamma = 1.0
Unsharp Masking	alpha = 1.5, beta = -0.5
Fourier Transform	-
Gabor Filter	kernel size = 41, sigma = 6.0, theta = $\pi/4$, lambda = 5.0, gamma = 0.9, psi = 0
Gradient Magnitude and Orientation	-

Table S2: Summary of image processing techniques and their parameters. These parameters were chosen from a mixture of visual inspection and documentation defaults. Overall our interests relate to having a range of values that match the categories of edge, contrast and noise variations - rather than how good the exact filtering system is.

To evaluate the performance of classifiers under datasets preprocess with each filter, we tested them on the textural datasets [1] described in the main paper. We made use of an LSTM of 13200 input nodes over 10 frames. The hidden layer had 50 nodes going through to 15 outputs. No activation function was used on the model. For training the learning rate was 5% with stochastic gradient descent (SGD) optimisation and cross entropy loss. We trained each model for 100 epochs 20 times with randomised weights and datasets.

**Method**	**Train Accuracy (%)**			**Test Accuracy (%)**		
	Mean	Max	Std	Mean	Max	Std
Gaussian Noise	27.01	34.58	3.49	23.67	34.33	3.68
Poisson Noise	35.28	44.16	5.37	31.11	42.49	5.55
Salt & Pepper Noise	42.61	51.12	3.76	38.87	46.16	4.44
Speckle Noise	57.93	79.45	9.41	54.90	78.32	10.68
Adaptive Histogram Equalization	80.12	96.41	8.87	78.70	95.98	8.97
Bilateral	58.19	74.21	9.13	56.04	71.49	9.08
Canny	**99.99**	99.99	0.01	**99.87**	99.98	0.13
Fourier Transform	0.00	0.00	0.00	0.00	0.00	0.00
Gabor Filter	**99.99**	99.99	0.00	**99.98**	99.98	0.00
Gamma Correction	77.58	89.87	9.25	75.99	89.49	10.35
Gaussian Blur	64.85	77.83	7.64	63.59	81.32	9.15
Histogram Equalization	83.28	96.08	6.14	82.30	96.98	6.26
Laplacian	32.63	53.58	7.57	28.27	49.66	7.26
Median Filter	74.06	96.45	10.38	73.30	96.65	10.99
Prewitt	54.29	77.83	9.74	51.03	76.65	10.57
Scharr	**99.99**	99.99	0.00	**99.96**	99.98	0.06
Sobel	**99.99**	99.99	0.01	**99.96**	99.98	0.06
Unsharp Masking	82.78	97.70	10.54	81.68	97.48	11.44
Wavelet Denoising	78.56	99.79	11.67	77.18	99.82	12.76
Compute Gradient Magnitude Orientation	**99.99**	99.99	0.00	**99.98**	99.98	0.04

Table S3: Train and Test Accuracy for Different Preprocessing Methods on the tactile dataset. Each model was trialled 20 times.

Table S3 shows the accuracy across the different filtering methods applied to the texture dataset. Filters that preserve the edge, and have little noise, perform higher than filters that are noisy. Contrast is irrelevant for the task of texture classification provided there is reduced noise and edge preservation.

1.4.2 PressTip

The PressTip has 16 channels, 3 used by an accelerometer (x, y, and z movements), 1 channel is from the piezoelectric sensor, and the remaining 12 are force sensitive resistors in an array of pressure pads. For the task of textural classification we make use of the vibration sensors (accelerometer and or piezoelectric) only. Pressure only detects weight distribution/orientation of the robot or shape of an environmental aspect such as an obstacle [2].

A number of signal processing patterns currently exist, but what aspects of this signal is actually impacting the ability for a model to detect what texture it is moving over? Existing literature has made use of time-domain techniques (which could include butterworth filter [3], low pass filter [4], Median filters [5], Savitzky–Golay filters [6]) [7], frequency-domain techniques (which cold include FFT [8], STFT [9], Mel-frequency cepstral coefficients [10], Power Spectral Density [11]) [12] and Time-frequency analysis (which could include Wavelet transforms [13], Hilbert-Huang transform [14]) [15].

To investigate the effectiveness of these filters on our electrical tactile sensor dataset [16] (consisting of 15 classes of texture) we applied them to the unprocessed dataset one by one and trained a random forest classifier across 40 trials (each RFC starting without a random state) to predict which texture the sensor is being dragged across. We chose to use a random forest regression classifier for its speed efficiency at training, compared to other models we trialled. The sensor was made up of accelerometers and piezoelectric sensors. We experimented across both sensors separately, and combined to see if there was an advantage to one over the other.

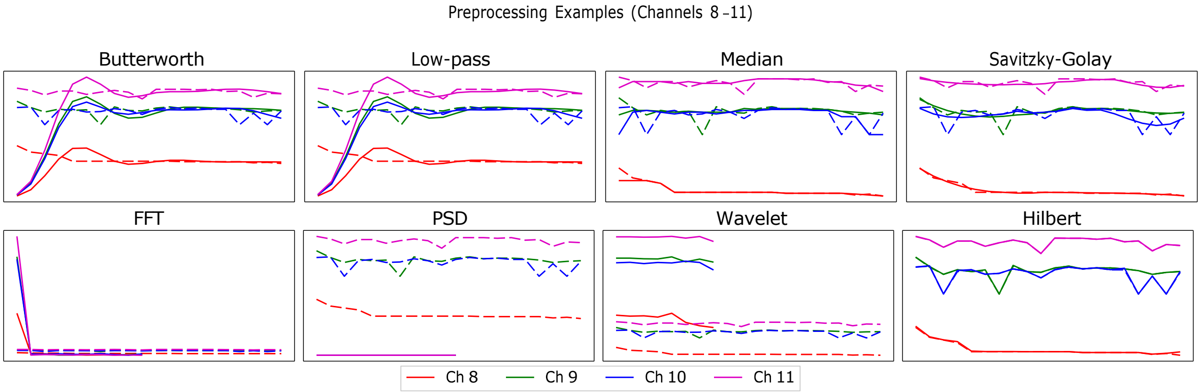

Figure S4: An example reading from the dataset is shown here. We show the piezoelectric (channel 8), accelerometer x (channel 9), accelerometer y (channel 10), and accelerometer z (channel 11). Shown using the dotted line, we display the original signal for each. In solid line, we show the processed signal. The x axis represents the recording index and y axis represents the voltage. This figure is to demonstrate examples of the influence on the signal, therefore axis are left unlabelled.

The influence of preprocessing highlights different characteristics of the tactile signals. Butterworth, low-pass, median, and Savitzky–Golay filters all act to smooth the signal, effectively reducing noise and removing spikes. FFT and PSD techniques reveal the frequency content of the signal, helping to identify texture-related vibration patterns. Wavelet transforms are sensitive to sharp changes and transitions within the signal, while the Hilbert transform emphasises the signal's envelope, capturing amplitude modulations over time. The results of these filters applied on a sample of the electrical signals is shown in Figure S4. The functional hyperparameters are outlined in Table S4. These values were chosen based on the sensor reading values, and documentation recommendations.

Table S4: Filter Methods and Their Hyperparameters

**Technique**	**Parameters**
Butterworth Filter	cutoff=8, fs=50, order=4
Low Pass Filter	cutoff=8, fs=50, order=4
Median Filter	kernel_size=5
Savitzky-Golay Filter	window_length=11, polyorder=3
FFT	None
Power Spectral Density (PSD)	fs=50, nperseg=min(64, len(d))
Wavelet Transform	wavelet='db4', level=3
Hilbert Transform	None
None	None

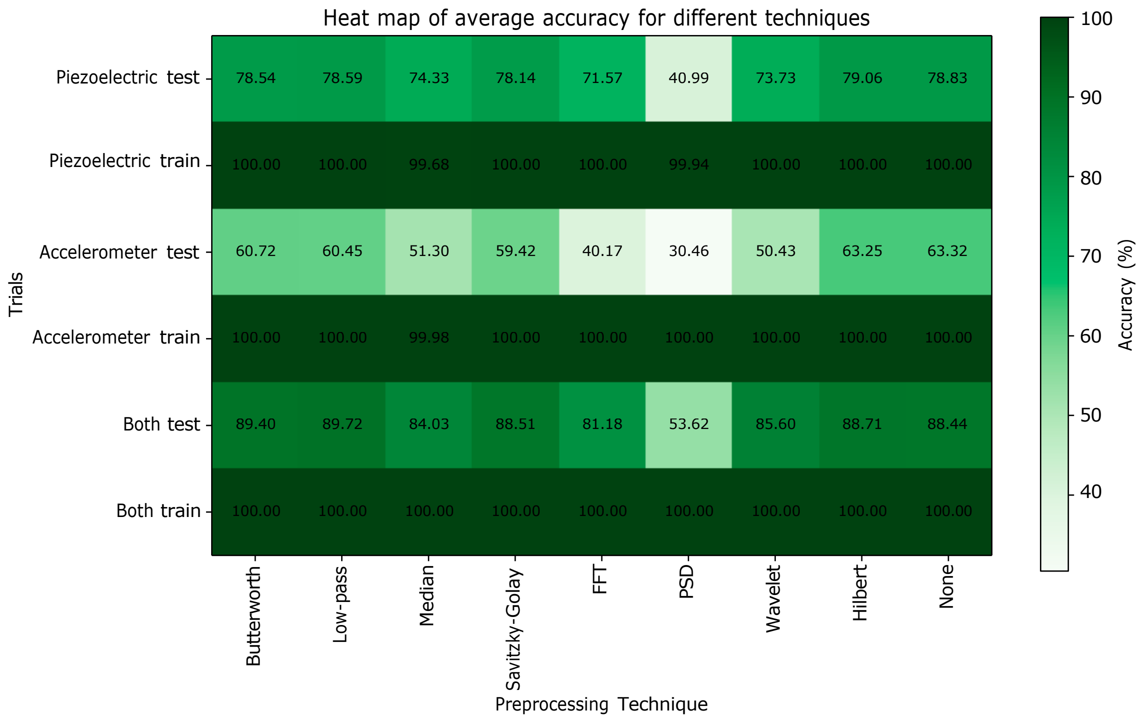

Figure S5: A heatmap of the test and train averaged accuracies over 40 trials for each of the preprocessing techniques.

The results from Figure S5 shows that there are higher accuracies mixing the sensor values than using them alone. In addition to this, the sensor does better on the signal smoothing approaches than on the others. This could be because the electrical noise is interfering with the sensor reading. We used the Wilcoxon rank-sum test with a p-value of 0.05. The Butterworth and Low pass are significantly higher than the other preprocessing techniques. The Butterworth and low-pass filter have no significance between them, either make a good candidate of preprocessing technique.

2 Additional Results and Analysis

2.1 Non-Linearity Study

Moving the sensor across varying vectors shows the sensor's ability to classify the different textures with linear stroking movements (as shown in the main paper). We also investigated how the sensor-classifier pairs performed on sensations they were not trained on. To do this, we collected a non-linear movement dataset by moving the sensor in circular movements of increasing radii (1cm, 1.75cm, 2.5cm) while increasing the pressure by approximately 17 grams on each iteration.

These datasets were preprocessed using the same techniques as the best performing model architecture previously trialled, as described in the main paper. The sensor-classifier pairs trained on the linear movement dataset, as detailed in the main paper, did not generalise to non-linear motion. The main paper focuses on developing a model that can distinguish the texture, however, for a wider range of potential applications, including in robotics, further investigations were carried out as detailed below.

One approach to allowing generalisation to non-linear movement, is to train classifiers on enough variations of direction and pressure that they become robust to non-linear movement. We have performed preliminary investigations of this approach by training classifiers for texture classification on a hybrid dataset comprising the non-linear data set described above and the original linear dataset described in the main paper, for both the TacTip and the PressTip. The approach was successful, achieving results comparable

with those for the linear dataset results as described in the main paper. Classifiers were able to generalise over both unseen linear and non-linear movement; see Table S5 below for the results.

**Sensor**	**Classifier**	**Test Accuracy**	**Train Accuracy**	**Std**
PressTip	RFC	77.3%	100%	0.00%
TACTIP	RFC	97.58%	98%	0.00%
PressTip	LSTM	57.8%	85.3%	0.011%
TACTIP	LSTM	83.71 %	84.9%	0.00%

Table S5: Comparison of PressTip and TACTIP texture classification performance trained on linear and non- linear data from 20 trials. The best performing classifier types from the previous linear dataset experiments were used.

Future work could involve incorporating SO(3) rotations to make the model invariant to orientation, thereby improving robustness to unseen sensory inputs. Or converting the images to a latent space using an autoecoder, to train models on a more compressed feature space. Making a fully generalisable model is a significant paper in its own right, and is outside the scope of the research described here. However, the preliminary results shown in Table S5 are very promising.

2.2 Model Training Times

We trained the models on a high performance computer with NVIDIA GeForce RTX 3090 (24GB GDDR6X) GPUS and 126GB of RAM. For CPU AMD Ryzen Threadripper 3970X, 32 cores / 64 threads. The server used Ubuntu 22.04.5 LTS (Jammy Jellyfish) with Kernel Linux 5.15.0-127-generic (64-bit).

The speed would vary depending on the size of the dataset. The model specifications are that of those used and described in the main paper. Because of the very different nature of the data for the optical TacTip and the electrical PressTip – the latter generally being much noisier – the speed of training for a particular classifier type could be very different between the two sensors.

**Sensor**	**Classifier**	**Time (roughly) hours**
TacTip	SVM	14
TacTip	RFC	0.49
TacTip	CNN	0.4
TacTip	LSTM	0.05
PressTip	SVM	0.015
PressTip	RFC	0.002
PressTip	ANN	8
PressTip	LSTM	9.2

Table S6: Models and their training times per model.

2.3 Selection of Friction Regression Model

The Random Forest Regression Classifier was found to be one of the quickest to train and highest performing for friction prediction, across both sensors. We performed a comparison to evaluate how the different classifiers performed for the task of friction prediction, using the metric of mean squared error. We compared the same models for both the electrical and optical sensor, provided the model type made sense for the type of data.

The LSTM and CNN classifiers both used hidden layers of 350 nodes. The neural models had one output node and were trained with the Adam optimizer with a learning rate of 0.001. The results are shown in Table S7. These results informed our decision to concentrate on The Random Forest Regression classifier in the friction prediction experiments described in the main paper.

Table S7: Model Performance Across Different Sensors averaged over 20 trials

**Model**	**Sensor**	**Test MSE**	**Train MSE**
Ridge regression	Optical	0.0002	0.0000
Ridge regression	Electrical	0.0238	0.0255
CNN	Optical	0.0412	0.0366
LSTM	Optical	0.0416	0.0368
LSTM	Electrical	0.0368	0.0361
Random Forest	Electrical	0.0030	0.0003
Random Forest	Optical	0.0002	0.0000

3 Source Code and Dataset

The source code and a sample dataset are available at:
Refined: https://github.com/shepai/Robot_foot
All code: https://github.com/shepai/RoboSkin
Kaggle datasets: https://www.kaggle.com/dextershepherd/datasets

References

[1] D. R. Shepherd. Optical tactile (tactip) dataset for texture classification, 2024. Available: https://doi.org/10.25377/sussex.26935696.

[2] D. R. Shepherd, P. Husbands, A. Philippides, and C. Johnson. Versatility of low-resolution tactile sensing for edge and pose detection. In *2024 5th International Conference on Artificial Intelligence, Robotics and Control (AIRC)*, pages 7–12. IEEE, 2024.

[3] Stephen Butterworth et al. On the theory of filter amplifiers. *Wireless Engineer*, 7(6):536–541, 1930.

[4] Lawrence C Wood. A review of digital pass filtering. *Reviews of Geophysics*, 6(1):73–97, 1968.

[5] Thomas S Huang. A fast two-dimensional median filtering algorithm, ieee trans. acout. speech. *Sig. Proces.*, 27:1, 1979.

[6] Ronald W Schafer. What is a savitzky-golay filter?[lecture notes]. *IEEE Signal processing magazine*, 28(4):111–117, 2011.

[7] Jeremy A Fishel and Gerald E Loeb. Bayesian exploration for intelligent identification of textures. *Frontiers in neurorobotics*, 6:4, 2012.

[8] Michael T Heideman, Don H Johnson, and C Sidney Burrus. Gauss and the history of the fast fourier transform. *Archive for history of exact sciences*, pages 265–277, 1985.

[9] Ervin Sejdi´c, Igor Djurovi´c, and Jin Jiang. Time–frequency feature representation using energy concentration: An overview of recent advances. *Digital signal processing*, 19(1):153–183, 2009.

[10] Min Xu, Ling-Yu Duan, Jianfei Cai, Liang-Tien Chia, Changsheng Xu, and Qi Tian. Hmm-based audio keyword generation. In *Pacific-Rim Conference on Multimedia*, pages 566–574. Springer, 2004.

[11] Alessandro Birolini. *Quality and reliability of technical systems: theory, practice, management.* Springer Science & Business Media, 2012.

[12] Shiyao Huang and Hao Wu. Texture recognition based on perception data from a bionic tactile sensor. *Sensors*, 21(15), 2021.

[13] Yves Meyer. *Wavelets and operators.* Number 37. Cambridge university press, 1992.

[14] Norden E Huang and Nii O Attoh-Okine. *The Hilbert-Huang transform in engineering.* CrC Press, 2005.

[15] Thiago Eustaquio Alves de Oliveira, Bruno Monteiro Rocha Lima, Ana-Maria Cretu, and Emil M Petriu. Tactile profile classification using a multimodal mems-based sensing module. In *Proceedings*, volume 1, page 27. MDPI, 2016.

[16] D. R. Shepherd. Electrical tactile dataset (piezoelectric and accelerometer) for textures, 2024. Available: https://doi.org/10.25377/sussex.28033589.
